# Supplementary material for: Modeling glioblastoma heterogeneity as a dynamic network of cell states
Source: Mol Syst Biol. 2021 Sep 16;17(9):e10105. doi: 10.15252/msb.202010105 (PMC8444284; doi:10.15252/msb.202010105)
Supplement: Supplementary file 5 — Source Data for Figure 3 [file MSB-17-e10105-s001.zip › Figure3A_sourcedata/GSEA_3065/nosigdb_state2.GseaPreranked.1622718419215/LARSSON_STATE_2.html]

Details for gene set LARSSON\_STATE\_2[GSEA]

|  || Dataset | state2 |
| Phenotype | NoPhenotypeAvailable |
| Upregulated in class | na\_neg |
| GeneSet | LARSSON\_STATE\_2 |
| Enrichment Score (ES) | -0.9114637 |
| Normalized Enrichment Score (NES) | -3.4073453 |
| Nominal p-value | 0.0 |
| FDR q-value | 0.0 |
| FWER p-Value | 0.0 |
Table: GSEA Results Summary

  

Fig 1: Enrichment plot: LARSSON\_STATE\_2      
 Profile of the Running ES Score & Positions of GeneSet Members on the Rank Ordered List

  

| PROBE | GENE SYMBOL | GENE\_TITLE | RANK IN GENE LIST | RANK METRIC SCORE | RUNNING ES | CORE ENRICHMENT || 1 | XRCC2 |  |  | 455 | 0.190 | -0.0396 | No |
| 2 | FUS |  |  | 6216 | -0.020 | -0.6543 | No |
| 3 | LAPTM4B |  |  | 7427 | -0.048 | -0.7813 | No |
| 4 | CREBZF |  |  | 7932 | -0.064 | -0.8321 | No |
| 5 | ARGLU1 |  |  | 8034 | -0.068 | -0.8397 | No |
| 6 | ZNF431 |  |  | 8628 | -0.105 | -0.8980 | No |
| 7 | NCAM1 |  |  | 8660 | -0.108 | -0.8962 | No |
| 8 | LUC7L3 |  |  | 8798 | -0.122 | -0.9050 | Yes |
| 9 | ANKRD11 |  |  | 8808 | -0.123 | -0.9001 | Yes |
| 10 | SF1 |  |  | 8915 | -0.137 | -0.9049 | Yes |
| 11 | PGRMC1 |  |  | 8958 | -0.143 | -0.9026 | Yes |
| 12 | SGCE |  |  | 8959 | -0.143 | -0.8958 | Yes |
| 13 | FGFBP3 |  |  | 8960 | -0.143 | -0.8890 | Yes |
| 14 | ZNF75A |  |  | 8980 | -0.146 | -0.8841 | Yes |
| 15 | PTN |  |  | 8989 | -0.147 | -0.8780 | Yes |
| 16 | PON2 |  |  | 9030 | -0.154 | -0.8749 | Yes |
| 17 | SLC1A3 |  |  | 9052 | -0.158 | -0.8696 | Yes |
| 18 | COL4A2 |  |  | 9069 | -0.161 | -0.8636 | Yes |
| 19 | PCSK7 |  |  | 9080 | -0.162 | -0.8570 | Yes |
| 20 | HACD3 |  |  | 9095 | -0.165 | -0.8506 | Yes |
| 21 | LRPAP1 |  |  | 9144 | -0.176 | -0.8473 | Yes |
| 22 | PLAT |  |  | 9145 | -0.176 | -0.8389 | Yes |
| 23 | SPCS2 |  |  | 9149 | -0.177 | -0.8308 | Yes |
| 24 | PRDX4 |  |  | 9151 | -0.178 | -0.8224 | Yes |
| 25 | MSMO1 |  |  | 9162 | -0.179 | -0.8150 | Yes |
| 26 | PDIA6 |  |  | 9165 | -0.180 | -0.8066 | Yes |
| 27 | ACSL3 |  |  | 9175 | -0.184 | -0.7988 | Yes |
| 28 | HMGCR |  |  | 9194 | -0.187 | -0.7918 | Yes |
| 29 | TTC14 |  |  | 9198 | -0.190 | -0.7831 | Yes |
| 30 | SCRG1 |  |  | 9200 | -0.191 | -0.7741 | Yes |
| 31 | SOX6 |  |  | 9203 | -0.192 | -0.7652 | Yes |
| 32 | SQLE |  |  | 9209 | -0.194 | -0.7564 | Yes |
| 33 | MDM4 |  |  | 9211 | -0.197 | -0.7472 | Yes |
| 34 | DDOST |  |  | 9213 | -0.198 | -0.7379 | Yes |
| 35 | LMAN1 |  |  | 9216 | -0.199 | -0.7286 | Yes |
| 36 | GRIA2 |  |  | 9222 | -0.202 | -0.7195 | Yes |
| 37 | MARCH6 |  |  | 9247 | -0.211 | -0.7120 | Yes |
| 38 | NKTR |  |  | 9259 | -0.216 | -0.7029 | Yes |
| 39 | DDX39B |  |  | 9263 | -0.219 | -0.6928 | Yes |
| 40 | TMBIM6 |  |  | 9264 | -0.219 | -0.6824 | Yes |
| 41 | ADGRG1 |  |  | 9275 | -0.222 | -0.6728 | Yes |
| 42 | CALU |  |  | 9278 | -0.223 | -0.6624 | Yes |
| 43 | SPPL2B |  |  | 9281 | -0.224 | -0.6519 | Yes |
| 44 | ZNF708 |  |  | 9283 | -0.225 | -0.6413 | Yes |
| 45 | COL4A1 |  |  | 9284 | -0.226 | -0.6305 | Yes |
| 46 | DDX17 |  |  | 9288 | -0.228 | -0.6200 | Yes |
| 47 | PRH1 |  |  | 9289 | -0.228 | -0.6091 | Yes |
| 48 | FTX |  |  | 9291 | -0.228 | -0.5984 | Yes |
| 49 | TMEM259 |  |  | 9292 | -0.228 | -0.5875 | Yes |
| 50 | LENG8 |  |  | 9296 | -0.229 | -0.5769 | Yes |
| 51 | PDIA3 |  |  | 9297 | -0.230 | -0.5660 | Yes |
| 52 | CALR |  |  | 9310 | -0.237 | -0.5560 | Yes |
| 53 | CD9 |  |  | 9324 | -0.248 | -0.5455 | Yes |
| 54 | GPC1 |  |  | 9326 | -0.250 | -0.5337 | Yes |
| 55 | RCN2 |  |  | 9332 | -0.255 | -0.5221 | Yes |
| 56 | ADGRV1 |  |  | 9333 | -0.256 | -0.5099 | Yes |
| 57 | HSP90B1 |  |  | 9334 | -0.257 | -0.4977 | Yes |
| 58 | KANSL1 |  |  | 9335 | -0.259 | -0.4854 | Yes |
| 59 | DDR1 |  |  | 9345 | -0.266 | -0.4736 | Yes |
| 60 | NOVA1 |  |  | 9348 | -0.272 | -0.4609 | Yes |
| 61 | PTPRZ1 |  |  | 9351 | -0.272 | -0.4481 | Yes |
| 62 | BCAN |  |  | 9353 | -0.276 | -0.4351 | Yes |
| 63 | SSR4 |  |  | 9359 | -0.287 | -0.4220 | Yes |
| 64 | ATP6AP2 |  |  | 9365 | -0.294 | -0.4085 | Yes |
| 65 | LAPTM4A |  |  | 9368 | -0.299 | -0.3944 | Yes |
| 66 | CANX |  |  | 9374 | -0.310 | -0.3802 | Yes |
| 67 | LAMP2 |  |  | 9380 | -0.322 | -0.3654 | Yes |
| 68 | GPM6A |  |  | 9381 | -0.324 | -0.3499 | Yes |
| 69 | B2M |  |  | 9383 | -0.331 | -0.3342 | Yes |
| 70 | MIAT |  |  | 9386 | -0.332 | -0.3186 | Yes |
| 71 | GPM6B |  |  | 9389 | -0.344 | -0.3025 | Yes |
| 72 | WSB1 |  |  | 9393 | -0.350 | -0.2861 | Yes |
| 73 | CD63 |  |  | 9394 | -0.353 | -0.2693 | Yes |
| 74 | MIR99AHG |  |  | 9395 | -0.354 | -0.2524 | Yes |
| 75 | SNTG1 |  |  | 9401 | -0.376 | -0.2351 | Yes |
| 76 | TNC |  |  | 9403 | -0.383 | -0.2169 | Yes |
| 77 | HSPA5 |  |  | 9406 | -0.395 | -0.1983 | Yes |
| 78 | APLP2 |  |  | 9409 | -0.408 | -0.1790 | Yes |
| 79 | APP |  |  | 9412 | -0.426 | -0.1589 | Yes |
| 80 | KCNQ1OT1 |  |  | 9414 | -0.434 | -0.1384 | Yes |
| 81 | MTRNR2L12 |  |  | 9416 | -0.436 | -0.1177 | Yes |
| 82 | VMP1 |  |  | 9417 | -0.448 | -0.0963 | Yes |
| 83 | MEG3 |  |  | 9419 | -0.461 | -0.0745 | Yes |
| 84 | MALAT1 |  |  | 9436 | -0.735 | -0.0412 | Yes |
| 85 | NEAT1 |  |  | 9439 | -0.871 | 0.0001 | Yes |
Table: GSEA details [plain text format]

  

Fig 2: LARSSON\_STATE\_2: Random ES distribution      
 Gene set null distribution of ES for **LARSSON\_STATE\_2**
